# Organo-Therapy in Ancient Times

**Published:** 1905-01-07

**Authors:** 


### Organo-Therapy in Ancient Times.

The use of drugs prepared from various animal tissues, which has so greatly extended in recent years, may be said to have had its counterpart in the dark ages of medicine. Two hundred years ago certain parts of the human body were believed to possess sovereign virtue as medicines, and were actually in use in the shops of the apothecaries. At the beginning of the eighteenth century the practice of using the component parts of the human body for therapeutic purposes had become less common than a century earlier. Still it was not extinct, and a reference to the medical books of the period reveals some rather gruesome beliefs and medicinal agents. We read, for instance, that the saliva of a healthy young man who had fasted for some time was remedial for the bites of serpents and mad dogs. For sufferers from hysterics the instructions were to burn the hair, so that the patients might inhale the fumes. The salt which ancient chemists distilled from human hair was used with supposed beneficial effects for many diseases of the brain, including epilepsy and apoplexy. An emetic was prepared by grating the nails of the fingers and toes, and administering in doses of one scruple. These cures are not nearly so repellant as some others that have had their day. For instance, in cases of epilepsy the human brain was given as an internal medicine. There were brains and brains, and all were not equally potent. A recommendation in a standard medical work of the time reads as follows :—"The brain should be drawn from the head of a healthy young man who has recently met with a violent death, as, for example, one who has been hanged but not yet interred." A liquid was usually drawn from the brain by distillation, but one authority claimed that a daily dose of two drachms of natural brain for twelve or fifteen days was much more efficacious than the liquid preparation. For general brain diseases the grated bones of human skulls were recognised remedies. The recommendations regarding the healthy young man newly killed, as recorded above, were given, and it was considered a mistake to reduce the bones to powder by calcination, which was thought to dispel the active properties in which lay the medicinal value.

Prepared human blood was another medicinal agent at one time in favour. The process of preparation consisted simply in allowing the blood to become hard in the sun and afterwards reducing it to powder in a mortar. It was given in doses of from one to two scruples in several kinds of fevers and in pleurisy.

The doctrine of *similia similibus curantur* was not overlooked, for stone in the bladder was supposed to be reduced by taking doses of stones from the bladders of men who had died from the disease—surely one of the most remarkable remedies ever suggested.

Finally, human fat was believed to be an excellent base for ointments, having the property of introducing the curative agents into the skin much better than other fats or oils.

In the search for medicinal remedies the belief seems to have been that the more extraordinary the substance the higher the virtue, and the tombs of Egypt contained objects too mysterious to be neglected. Thus there existed several preparations of Egyptian mummy. The rules to be observed to obtain medicines of high potency were as follows :—"Choose a mummy clean, beautiful, black, and glossy, with a smell sufficiently strong but not disagreeable." Preparations of mummy were believed to be tonic in their effects, resisting gangrene, and were considered as excellent applications for wounds.

